# Nationwide characteristics of infectious disease patients transported by helicopter ambulance in Türkiye

**DOI:** 10.55730/1300-0144.6369

**Published:** 2026-06-17

**Authors:** Ayşe Gülden BEKGÖZ, Kerem Dost BİLMEZ, Muhammed Saltuk DENİZ

**Affiliations:** 1 Department of Infectious Diseases and Clinical Microbiology, Ankara Gaziler Physical Therapy and Rehabilitation Training and Research Hospital, Ankara, Turkiye; 2 General Directorate of Emergency Health Services, Ministry of Health of the Republic of Türkiye, Ankara, Turkiye; 3 Department of Emergency Medicine, Ankara Training and Research Hospital, Ankara, Turkiye

**Keywords:** Air ambulance, emergency medical services, helicopter ambulance, infectious diseases, interhospital transfer

## Abstract

**Background/aim:**

To evaluate the clinical, demographic, and operational characteristics of patients with infectious diseases transported by helicopter ambulance and to examine differences in the transfer patterns, flight durations, and diagnostic categories.

**Materials and methods:**

Registry data of patients with infectious diseases transported by helicopter ambulance were analyzed. Demographic characteristics, diagnoses, referring and receiving hospital types, intraprovincial and interprovincial transfer status, flight durations, and temporal distribution of transfers (hours, days, and months) were evaluated. Flight duration was calculated in minutes and compared between intraprovincial and interprovincial transfers.

**Results:**

A total of 543 patients with infectious diseases were transported by helicopter ambulance. Respiratory tract infections were the most common diagnostic group, followed by coronavirus disease 2019. Of the patients, 72.9% were transported within the same province, while 27.1% were transferred between provinces. The overall median helicopter flight duration was 63 min (interquartile range (IQR): 45–87). Interprovincial transfers had significantly longer flight durations compared with intraprovincial transfers (median: 115 min (IQR: 63–222.5) vs. 57 min (IQR: 41–71); p < 0.001). In multivariable analysis, patients diagnosed with meningoencephalitis were more likely to be transferred to interprovincial centers (odds ratio (OR): 4.77; 95% confidence interval (CI): 1.26–18.07), whereas patients with sepsis (OR: 0.11; 95% CI: 0.03–0.48) and skin and soft tissue infections (OR: 0.08; 95% CI: 0.01–0.93) were more commonly managed with intraprovincial transfers.

**Conclusion:**

The findings indicate that helicopter ambulance use for patients with infectious diseases is applied through a selective and needs-based approach. In time-critical and high-risk infections such as meningoencephalitis, helicopter ambulance transport appears to be an important component of the healthcare system by facilitating access to appropriate referral centers. Differences between intraprovincial and interprovincial transfers suggest that air ambulance utilization is determined not only by distance and geography but also by the need for specialized care and advanced treatment capabilities.

## 1. Introduction

Emergency medical services (EMS) play a critical role in saving lives by providing rapid medical intervention and safe patient transport in out-of-hospital settings. Traditionally, ground EMS (GEMS) represent the primary mode of emergency response delivered via ambulances. However, in time-sensitive situations and in regions with limited accessibility, helicopter EMS (HEMS) has emerged as an effective alternative. HEMS accelerates access to healthcare services, particularly in trauma, cardiac events, and other acute medical conditions, thereby reducing mortality [1]. Geographic challenges, transport time, and patient safety are key determinants in emergency transfer system planning. In countries with large surface areas and heterogeneous topography, the timely transport of critically ill patients to adequately equipped centers is of vital importance for patient prognosis [2]. In time-dependent conditions such as trauma or cardiovascular diseases, the use of helicopter ambulances can significantly shorten rescue times and improve patient outcomes [3,4]. Nevertheless, factors such as flight distance, meteorological conditions, patient stabilization, and pretransport preparation are important variables influencing operational success. Although the literature reports that interhospital helicopter transfers significantly reduce time to reach the destination center in time-sensitive cases and enable earlier access to advanced life support, the risks of complications, costs, and logistical constraints remain subjects of ongoing debate [1,3,5,6].

In Türkiye, the air ambulance system was initiated on October 28, 2008, with two helicopters and was expanded nationwide in 2009. Currently, 13 helicopter ambulances are stationed in 13 provinces, positioned based on geographic structure, population density, and meteorological conditions. These helicopters operate between sunrise and sunset and are capable of transporting patients over distances of up to approximately 400 km without refueling; each mission is staffed by one physician, one healthcare professional, and two pilots. Operations are coordinated by the Provincial Health Command and Control Centers and the Ministry of Health Air Operations Center. This system enables the rapid and safe transfer of critically ill patients, particularly from remote regions^1^. While fixed-wing air ambulances provide advantages in long-distance transfers, helicopter ambulances are more effective in short-range emergency situations [7]. In a comprehensive analysis conducted in Türkiye, 4248 helicopter missions were evaluated, revealing that most transports were related to cardiovascular diseases (39.5%), trauma (15.4%), and neurological disorders (12.9%), with a mean flight time of 35.5 ± 23.3 min [8].

Although HEMS has been extensively studied, research specifically focusing on patient subgroups with infectious diseases transported by helicopter ambulance remains limited. In Türkiye, previous research has predominantly addressed trauma, cardiovascular emergencies, and general internal medicine cases, while the epidemiology, logistical characteristics, and clinical profiles of infectious disease-related air medical transfers have not been adequately explored [8–10]. During the pandemic period, studies on the helicopter transport of patients with coronavirus disease 2019 (COVID-19) demonstrated the potential role of air ambulance systems in combating infectious diseases; it was reported that the use of negative-pressure isolation units became more widespread during this period and that delays in the interhospital transfer of critically ill patients could adversely affect the administration of antimicrobial therapy [9–11].

Infectious diseases encompass a broad spectrum of high-risk and time-critical conditions, such as sepsis, meningitis, viral encephalitis, hemorrhagic fever syndromes, and severe viral pneumonias, all of which are associated with substantial morbidity and mortality. In these conditions, early diagnosis, appropriate isolation measures, and rapid transfer to suitably equipped referral centers are crucial determinants of prognosis. Therefore, a detailed evaluation of the clinical and operational characteristics of infectious disease–related transfers conducted by helicopter ambulance may provide valuable insights and contribute to the limited existing literature in this field.

Accordingly, the present study aimed to describe the demographic, clinical, and operational characteristics of adult patients with infectious diseases transported by HEMS across Türkiye and to evaluate the relationships between diagnostic categories and transfer parameters.

## 2. Materials and methods

This retrospective, descriptive study included adult patients diagnosed with infectious diseases who were transported by helicopter ambulance across Türkiye between January 1, 2020, and December 31, 2024. The study was approved by the Ethics Committee of the Ankara Bilkent City Hospital Medical Research Ethics Committee (Approval No: TABED 2-25-1578).

The data were obtained from the Emergency Health Automation System (EHAS) of the General Directorate of Emergency Health Services, Ministry of Health. EHAS is a nationwide electronic registry system operating on secure servers and is closed to external access. The system is routinely used for recording all EMS missions across Türkiye. The data are entered by authorized EMS personnel using individually assigned usernames and passwords. Once entered, records cannot be modified, ensuring data integrity and consistency. Due to its secure infrastructure and controlled access, EHAS represents a reliable and standardized source of nationwide EMS data.

Adult patients aged 18 years and older who were transported by HEMS with a diagnosis of an infectious disease were included in the analysis. Records with missing or duplicate data, as well as pediatric cases, were excluded from the study. Demographic variables (age and sex), clinical diagnosis, types of referring and receiving centers, and operational variables such as flight time and mission timing were evaluated.

Infectious disease diagnoses were defined based on the International Classification of Diseases, 10th Revision (ICD-10) codes in combination with available clinical information recorded in the system. These diagnoses were established at the referring hospitals prior to transport, and the HEMS team did not perform diagnostic evaluations but transported patients based on preexisting diagnoses. COVID-19 was evaluated as a separate diagnostic category, as it represents a systemic infectious disease that may affect multiple organ systems and is not limited to the respiratory tract.

Flight time was calculated in minutes and defined as the total duration of the helicopter mission.

### 2.1. Statistical analysis

Statistical analyses were performed using Microsoft Excel (Microsoft Corp., Redmond, WA, USA) and IBM SPSS Statistics for Windows, Version 20.0 (IBM Corp., Armonk, NY, USA). Continuous variables were expressed as mean ± standard deviation (SD) or median and interquartile range (IQR). Categorical variables were presented as frequencies and percentages. The normality of continuous variables was assessed using the Kolmogorov–Smirnov test. Comparisons between groups were performed using the Student’s t test or Mann–Whitney U test for continuous variables and the chi-squared test or Fisher’s exact test for categorical variables.

Univariable and multivariable logistic regression analyses were conducted to identify factors associated with interprovincial helicopter ambulance transfer. Univariable analysis included age, sex, reasons for transfer, referring and receiving hospital types, and infectious disease categories. Variables considered clinically relevant were included in the multivariable logistic regression model to ensure a comprehensive evaluation of associated factors. p < 0.05 was considered statistically significant.

## 3. Results

Between January 1, 2020, and December 31, 2024, a total of 15,060 helicopter ambulance transport records were reviewed across Türkiye. Based on ICD diagnosis names, 829 patients with potentially infection-related diagnoses were identified. Of these, 207 were younger than 18 years, 56 had missing data, and 23 were duplicate records; therefore, they were excluded. A total of 543 patients (3.6%) were included in the final analysis. The mean age of the patients was 63.1 ± 19.9 years, and 51.7% were male. Respiratory tract infections constituted the most common diagnostic group, accounting for 33.5% (n = 182) of all cases. This group included pneumonia, bronchitis, lung abscess, and infection-related chronic obstructive pulmonary disease (COPD) exacerbations. COVID-19 was the second most frequent diagnosis, observed in 25.7% (n = 140) of patients. Hepatobiliary and intraabdominal infections accounted for 16.8% (n = 91) of cases. Other less frequent diagnoses included sepsis (7.0%), acute gastroenteritis (2.8%), myocarditis/endocarditis/pericarditis (2.6%), meningoencephalitis (2.6%), hepatitis (2.0%), and skin and soft tissue infections (1.8%).

The mean body temperature of the patients was 36.7 ± 0.7 °C. The mean systolic and diastolic blood pressures were 118.5 ± 20.4 and 72.1 ± 12.6 mmHg, respectively. The median Glasgow Coma Scale score was 15 [13–15] (Table 1).

The most common indication for helicopter ambulance transfer was the need for a specialist physician (62.6%), followed by the need for intensive care (23.2%) and a lack of treatment or equipment (7.4%). The majority of patients were referred from state hospitals (84.7%). Regarding receiving centers, most patients were transferred to training and research hospitals (51.4%) and university hospitals (23.9%) (Table 2).

Of the 543 patients, 396 (72.9%) were transported within the same province, whereas 147 patients (27.1%) were transported between different provinces. The overall median flight time was 63 min (IQR: 45–87). Flight time was significantly longer in interprovincial transfers than in intraprovincial transfers (median: 115 min (IQR: 63–222.5) vs. 57 min (IQR: 41–71); p < 0.001).

**Figure 1:**
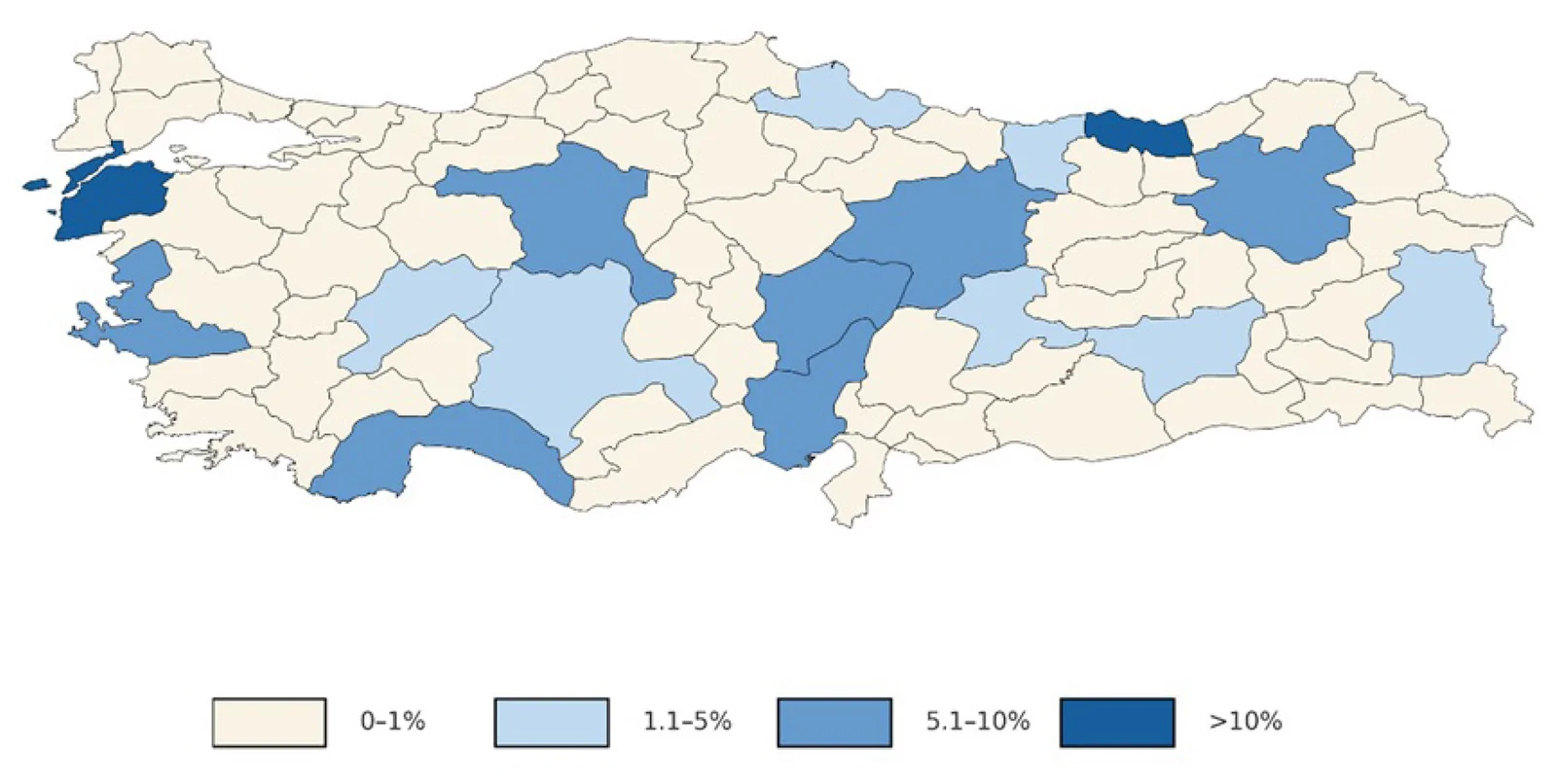
Distribution of intraprovincial helicopter ambulance transfers among patients with infectious diseases.

When the provincial distribution was examined, the highest proportions of intraprovincial helicopter ambulance transfers were observed in Trabzon (18.2%) and Çanakkale (10.9%). Provinces with rates between 5% and 10% included İzmir (8.3%), Ankara (7.1%), Adana (6.8%), Sivas and Kayseri (6.3%), Antalya (5.8%), and Erzurum (5.6%). Provinces with rates between 1% and 5% were Diyarbakır (4.8%), Samsun and Malatya (3.8%), Van (3.5%), Konya (2.5%), Giresun (2.3%), and Afyon (1.8%). Rates between 0% and 1% were observed in Bursa and İstanbul (0.8%), as well as Elazığ, Manisa, and Hakkari (0.3%) (Figure 1).

Of the patients diagnosed with infectious diseases, 147 (27.1%) were transported by helicopter ambulance between provinces. Among interprovincial transfers, the most frequent referring provinces were Trabzon (11.6%), Çanakkale (9.5%), Şırnak (6.1%), and Sivas (5.4%).

**Figure 2:**
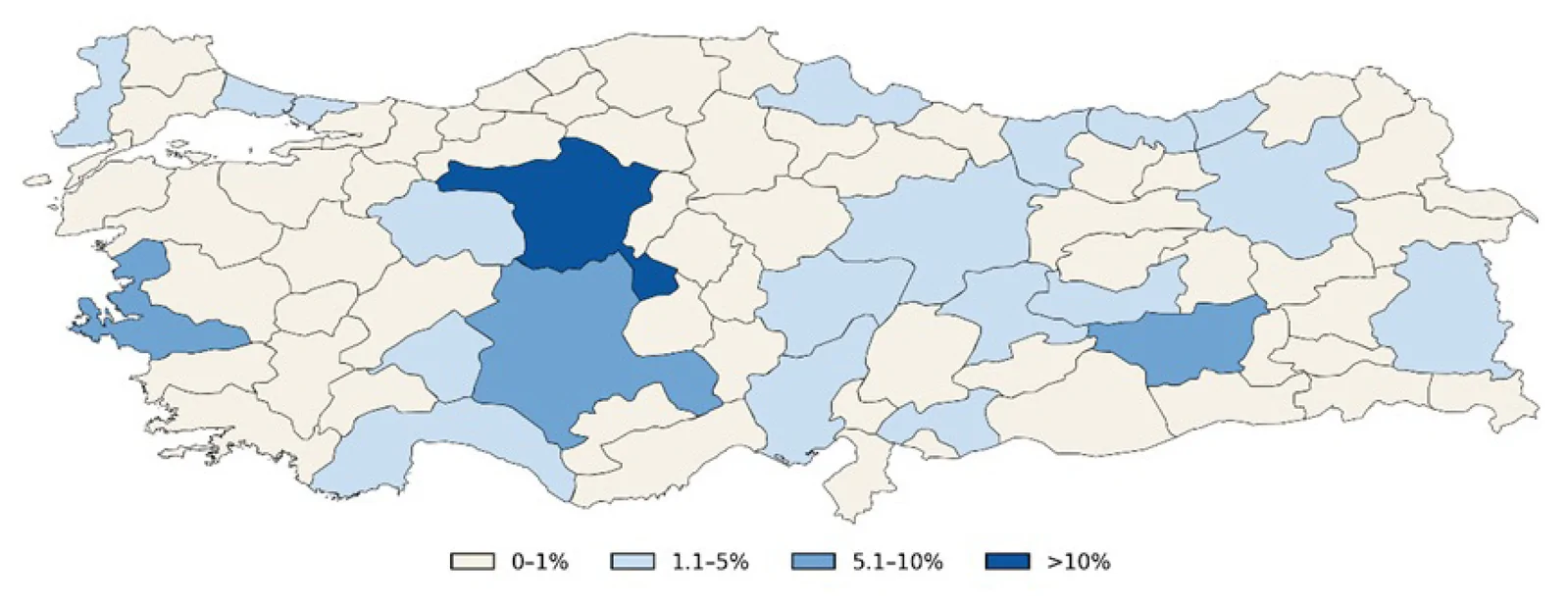
Distribution of receiving provinces for interprovincial helicopter ambulance transfers among patients with infectious diseases.

When the receiving provinces were examined, patients were most frequently transferred to centers in Ankara (25.2%). This was followed by İzmir (8.2%), and Konya and Diyarbakır (5.4% each). Other provinces, including Isparta, İstanbul, Samsun, Malatya, and Edirne, each received between 1.1% and 5.0% of patients transported by interprovincial helicopter ambulance (Figure 2).

In the multivariable logistic regression analysis conducted to identify factors associated with interprovincial helicopter ambulance transfer, age and sex were not independently associated with interprovincial transfer. When reasons for transfer were evaluated, the need for intensive care (odds ratio (OR) 4.28; 95% confidence interval (CI): 2.32–7.92; p < 0.001), lack of treatment or equipment (OR: 3.65; 95% CI: 1.47–9.08; p = 0.005), transfer to the center where the patient was previously followed up (OR: 19.14; 95% CI: 4.29–85.31; p < 0.001), and the need for surgery or transplantation (OR: 16.67; 95% CI: 2.83–98.05; p = 0.002) were independently associated with interprovincial helicopter ambulance transfer.

With respect to infectious disease categories, patients diagnosed with meningoencephalitis were more frequently transferred by interprovincial helicopter ambulance (OR: 4.77; 95% CI: 1.26–18.07; p = 0.021), whereas patients diagnosed with sepsis (OR: 0.11; 95% CI: 0.03–0.48; p = 0.003) and skin and soft tissue infections (OR: 0.08; 95% CI: 0.01–0.93; p = 0.044) were predominantly managed with intraprovincial transfers.

When the type of referring hospital was examined, transfers originating from training and research hospitals (OR: 32.40; 95% CI: 8.82–119.08; p < 0.001), private hospitals (OR: 55.49; 95% CI: 12.25–251.35; p < 0.001), and university hospitals (OR: 27.73; 95% CI: 6.40–120.19; p < 0.001) were strongly and independently associated with interprovincial helicopter ambulance transfer compared with transfers from state hospitals (Table 3).

**Figure 3:**
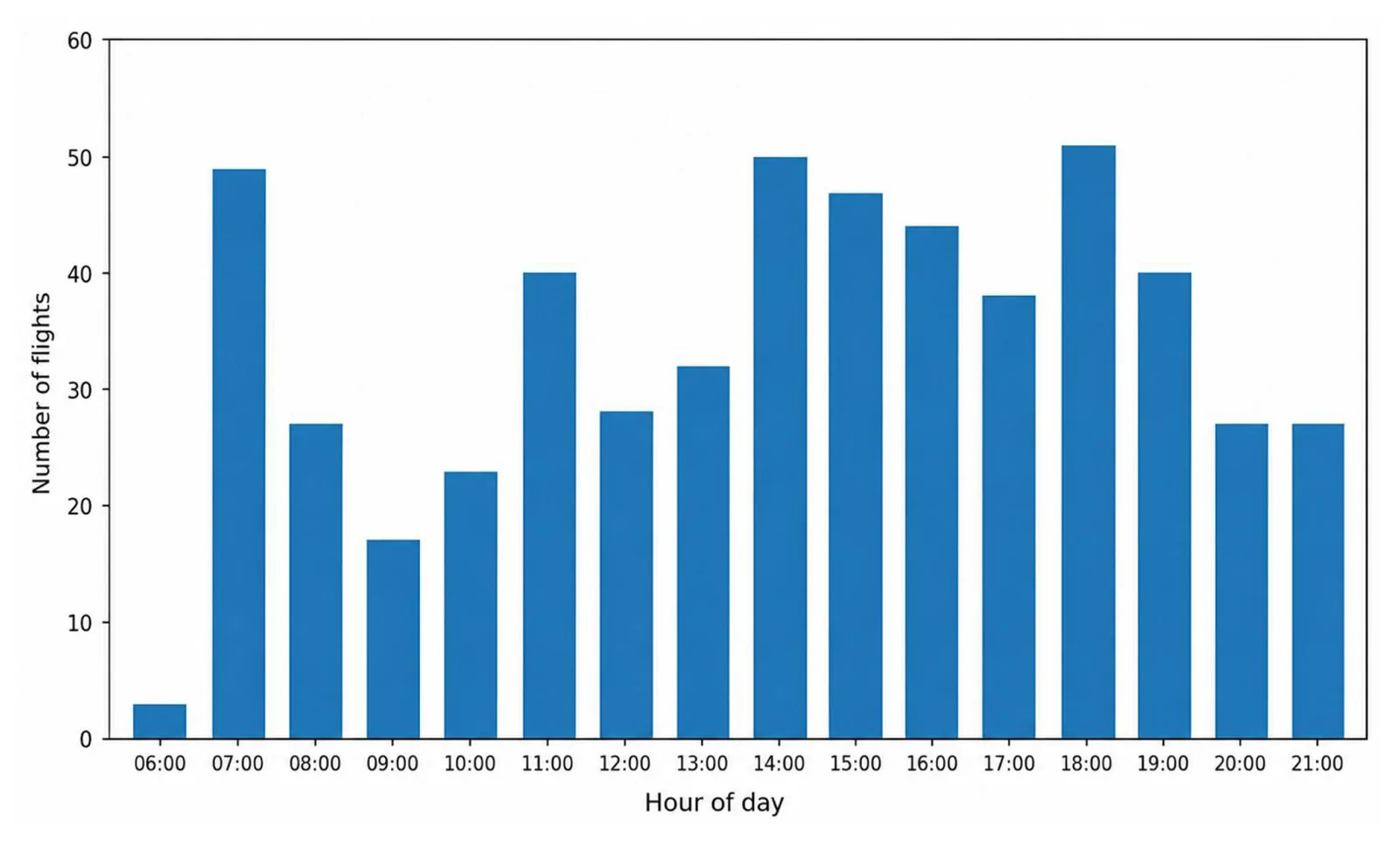
Hourly distribution of helicopter ambulance flights for patients with infectious diseases.

When the hourly distribution of helicopter ambulance transfers for patients with infectious diseases was analyzed, flights were found to be concentrated during specific hours of the day. The highest number of flights occurred between 14:00 and 18:00 h. A statistically significant difference was observed in the hourly distribution of flights (chi-squared test: 79.11, p < 0.001). The hourly distribution of flights is presented in Figure 3.

Of all cases, 74.8% occurred on weekdays and 25.2% on weekends. No statistically significant difference was found in case frequency between weekdays and weekends (p > 0.05).

In the seasonal analysis, 31.5% of cases occurred in autumn, followed by spring (28.2%), winter (22.3%), and summer (18.0%).

## 4. Discussion

This nationwide study provides a comprehensive evaluation of adult patients with infectious diseases transported by helicopter ambulance in Türkiye. Infectious diseases constituted a small but clinically distinct subgroup of air ambulance transport, with respiratory tract infections and COVID-19 being the most common indications. Overall, the findings suggest that air ambulance utilization in infectious diseases is shaped not only by disease severity but also by the need for specialized care and healthcare system organization.

Although most transfers were conducted within the same province, a substantial proportion involved interprovincial transport, suggesting variability in local healthcare capacity and access to specialized care. The higher frequency of interprovincial transfers among patients requiring intensive care and those with conditions such as meningoencephalitis likely reflects the need for advanced diagnostic and therapeutic resources available in tertiary centers. In contrast, the predominance of intraprovincial transfers among patients with sepsis and skin and soft tissue infections suggests that these conditions can often be managed within existing local healthcare infrastructure. Furthermore, differences in transfer patterns according to referring and receiving hospital types highlight the influence of healthcare system organization on air ambulance utilization.

The prominence of respiratory tract infections among air-transported infectious disease patients may be related to the high frequency of severe respiratory compromise requiring advanced respiratory support. Previous studies have predominantly focused on time-sensitive conditions such as trauma, cardiovascular emergencies, and stroke, whereas infectious diseases have remained a relatively underexplored area in the context of helicopter ambulance services [12]. This observation is consistent with previous reports indicating that a substantial proportion of infectious disease cases transported by both helicopter and fixed-wing air ambulances involve conditions such as pneumonia and severe respiratory failure requiring advanced airway management and mechanical ventilation [10,13]. Overall, the predominance of respiratory system-related conditions among air-transported infectious disease patients suggests that severe respiratory involvement is a key determinant of air ambulance use. The high proportion of COVID-19 cases is consistent with both its potential to cause significant respiratory compromise and the inclusion of pandemic years within the study period. In addition, the notable representation of hepatobiliary and intraabdominal infections may reflect the need for referral to higher-level centers due to increased requirements for advanced diagnostic and interventional procedures as well as intensive care support.

Transfer indications in patients with infectious diseases suggest that air ambulance utilization is not determined solely by disease severity but is also closely related to the need for specialist evaluation and access to higher-level healthcare services. The prominence of specialist consultation as a transfer indication highlights the role of helicopter ambulance services in facilitating timely referral to tertiary centers, particularly in complex cases requiring multidisciplinary management, including infectious diseases, intensive care, and interventional specialties.

Similarly, previous national studies have shown that interhospital transfers conducted by ground ambulance are not determined solely by disease severity but are also influenced by the need for specialist evaluation and access to advanced diagnostic and therapeutic facilities [14]. Although studies specifically addressing interhospital transfer indications in patients with infectious diseases are limited, the literature on interhospital transport of critically ill and intensive care patients suggests that transfer decisions are not based solely on diagnostic categories. Instead, these decisions are influenced by both clinical and organizational factors. Clinical considerations include patient stability, the need for intensive care, requirements for advanced monitoring and treatment, and potential risks during transport, whereas organizational factors involve team expertise, the availability of appropriate equipment, and logistical planning [15,16].

In this context, the predominance of transfers originating from state hospitals and being directed to training and research hospitals and university hospitals suggests that the air ambulance system plays a complementary role in addressing differences in expertise and infrastructure between peripheral healthcare facilities and tertiary referral centers.

The predominance of intraprovincial transfers among patients with infectious diseases suggests that, in many cases, access to advanced healthcare services is available within provincial boundaries. However, the clustering of intraprovincial transfers in certain provinces indicates that this pattern may be influenced not only by healthcare system organization but also by regional geographical characteristics. In particular, challenging terrain and limitations in road transportation, especially in regions such as the Black Sea area, may complicate patient transfer even within the same province, making helicopter ambulance transport a practical and facilitative option. In contrast, interprovincial transfers may reflect situations in which infectious disease presentations exceed local healthcare capacity, requiring access to advanced subspecialty care, intensive care resources, or interventional treatments. Similarly, international studies have shown that non-trauma use of HEMS is more prominent in settings characterized by geographical accessibility challenges and the need for tertiary-level healthcare services [17,18]. These findings suggest that helicopter ambulance use in patients with infectious diseases is influenced not only by distance but also by geographical conditions and the need for access to higher-level healthcare services.

The temporal distribution of helicopter ambulance transfers in patients with infectious diseases suggests that air ambulance utilization should be evaluated not only in terms of clinical urgency but also within the context of the daily and weekly organization of healthcare services. The predominance of weekday transfers may reflect more structured and systematically functioning healthcare delivery processes during these periods. Although transfers occurred throughout the day, the observed increase during afternoon hours suggests that helicopter ambulance use may concentrate within specific time intervals in relation to operational planning and referral processes. In Türkiye, helicopter ambulance operations are primarily conducted between sunrise and sunset, which may influence flight planning and transfer timing. While interhospital transfers by ground ambulance can be performed at any time of day, the restriction of helicopter operations to daylight hours suggests that air ambulance referral and scheduling are organized within defined operational time frames and constraints [14].

The seasonal distribution of transfers, with higher frequencies observed in autumn and spring, is consistent with known epidemiological patterns of infectious diseases. Respiratory tract infections exhibit seasonal variation, with influenza peaking in the autumn–winter period, respiratory syncytial virus (RSV) in winter, and other viral pathogens showing variable seasonal trends [19,20]. This pattern may be related to seasonal increases in infectious diseases and variations in healthcare system burden across different periods of the year.

The temporal distribution of helicopter ambulance missions has been shown to be closely related to operational planning and healthcare system functioning. A single-center study from Türkiye similarly demonstrated that the clinical characteristics and outcomes of patients transported by helicopter ambulance were associated with referral processes and operational factors [21]. Similarly, a study conducted in rural settings showed that the timing of HEMS missions is influenced by geographical accessibility, case type, and operational conditions [22]. The temporal distribution observed in this study, consistent with previous findings, suggests that helicopter ambulance utilization is shaped not only by case-related requirements but also by operational planning and accessibility conditions.

In this context, current international guidelines for the management of acute meningoencephalitis emphasize that early diagnosis, rapid initiation of antimicrobial therapy, access to advanced imaging modalities, and close neurological monitoring are critical determinants of prognosis^2^. The more frequent referral of patients with meningoencephalitis to interprovincial centers appears to be consistent with the need for advanced infrastructure and a multidisciplinary approach during diagnosis and treatment.

Similarly, the literature on sepsis management highlights that early recognition, timely initiation of antimicrobial therapy, and appropriate intensive care support are key determinants of mortality [23]. The predominance of intraprovincial referrals among patients with sepsis suggests that rapid intervention using existing local resources may be prioritized over interprovincial transfer.

For skin and soft tissue infections, most cases can be effectively managed at local centers with appropriate medical and surgical treatment. However, the literature indicates that only severe and rapidly progressive conditions, such as necrotizing fasciitis, benefit from early surgical debridement and intensive care support in terms of reduced mortality [24]. The predominance of intraprovincial transfers further supports the conclusion that these conditions can generally be managed at local hospitals.

In addition, the existing literature on helicopter ambulance utilization has largely focused on trauma and cardiovascular emergencies, whereas studies addressing infectious diseases—particularly those evaluating clinical subgroups in relation to transfer patterns—remain limited [18,22,25]. Therefore, although direct comparison with previous studies is challenging, our findings provide a unique contribution by identifying which groups of infectious disease patients are selectively prioritized in nontrauma HEMS utilization.

This study had several limitations. Due to its retrospective design based on registry data, clinical outcomes such as mortality, length of hospital stay, and functional outcomes could not be evaluated. In addition, the lack of standardized and complete prehospital disease severity scores—such as the Sequential Organ Failure Assessment (SOFA), qSOFA, or similar scoring systems—for all cases precluded risk-adjusted analyses. The inclusion of data from a single national air ambulance system may also limit the generalizability of the findings to other healthcare systems. Nevertheless, the evaluation of a large, nationwide patient cohort provides an important framework for understanding the clinical and operational characteristics of infectious disease patients transported by helicopter ambulance.

## 5. Conclusion

Helicopter ambulance utilization in patients with infectious diseases is shaped by a complex interplay of clinical urgency, healthcare system capacity, and organizational factors rather than disease severity alone. Air medical transport serves a critical and strategic function in ensuring timely access to specialized care, particularly for complex and time-sensitive conditions requiring advanced infrastructure and multidisciplinary management.

These findings underscore the need to integrate both clinical and system-level determinants into decision-making processes and highlight the importance of optimizing air ambulance planning and resource allocation. By providing nationwide data, this study contributes to a better understanding of non-trauma air medical transport and offers a framework for improving the efficiency and appropriateness of helicopter ambulance utilization in infectious diseases.

## Figures and Tables

**Table 1:** Demographic, clinical, and diagnostic characteristics of patients transported by helicopter ambulance.

Variable	Value

**Sex**	
Male	281 (51.7%)
Female	262 (48.3%)

**Age, years (mean ± SD)**	63.1 ± 19.9

**Glasgow Coma Scale score (median [min–max])**	15 [13–15]

**Body temperature, °C (mean ± SD)**	36.7 ± 0.7

**Arterial blood pressure, mmHg (mean ± SD)**	
Systolic	118.5 ± 20.4
Diastolic	72.1 ± 12.6

**Primary diagnosis**	
Respiratory tract infections (pneumonia, bronchitis, lung abscess, COPD exacerbation)	182 (33.5%)
COVID-19	140 (25.7%)
Hepatobiliary and intraabdominal infections	91 (16.8%)
Sepsis	38 (7.0%)
Acute gastroenteritis	15 (2.8%)
Myocarditis/endocarditis/pericarditis	14 (2.6%)
Meningoencephalitis	14 (2.6%)
Hepatitis (acute or reactivation)	11 (2.0%)
Skin and soft tissue infections	10 (1.8%)
Other infections*	28 (5.2%)

* Other infections include thrombophlebitis, Crimean–Congo hemorrhagic fever, urinary tract infections, fever of unknown origin, and prion disease.

**Table 2:** Reasons for transfer and types of referring and receiving hospitals among patients transported by helicopter ambulance.

Variable	Value

**Reason for transfer**	
Need for specialist physician	340 (62.6%)
Need for intensive care	126 (23.2%)
Lack of treatment/equipment	40 (7.4%)
Transfer to follow-up center	24 (4.4%)
Need for surgery/transplantation	13 (2.4%)

**Hospital referred from**	
State hospital	460 (84.7%)
Training and research hospital	35 (6.5%)
University hospital	22 (4.1%)
Private hospital	26 (4.7%)

**Hospital transported to**	
Training and research hospital	279 (51.4%)
University hospital	130 (23.9%)
State hospital	89 (16.4%)
Private hospital	45 (8.3%)

**Table 3:** Multivariable logistic regression analysis of factors associated with interprovincial helicopter ambulance transfer.

Variable	OR (95% CI)	p-value

**Demographic factors**		
Age (years)	0.99 (0.98–1.00)	0.218
Male sex	0.97 (0.57–1.65)	0.908

**Reason for transfer**		
Need for specialist physician	Reference	—
Need for intensive care	**4.28 (2.32–7.92)**	**<0.001**
Lack of treatment/equipment	**3.65 (1.47–9.08)**	**0.005**
Transfer to follow-up center	**19.14 (4.29–85.31)**	**<0.001**
Need for surgery/transplantation	**16.67 (2.83–98.05)**	**0.002**

**Infectious disease categories**		
Respiratory tract infections	Reference	—
COVID-19	0.70 (0.36–1.36)	0.295
Hepatobiliary/intraabdominal infections	0.79 (0.34–1.82)	0.576
Sepsis	**0.11 (0.03–0.48)**	**0.003**
Acute gastroenteritis	0.72 (0.14–3.59)	0.689
Myocarditis	0.15 (0.01–2.52)	0.186
Meningoencephalitis	**4.77 (1.26–18.07)**	**0.021**
Hepatitis	3.49 (0.62–19.56)	0.155
Skin and soft tissue infections	**0.08 (0.01–0.93)**	**0.044**
Other infections^*^	1.64 (0.54–4.93)	0.381

**Hospital referred from**		
State hospital	Reference	—
Training and research hospital	**32.40 (8.82–119.08)**	**<0.001**
Private hospital	**55.49 (12.25–251.35)**	**<0.001**
University hospital	**27.73 (6.40–120.19)**	**<0.001**

Bold values indicate statistically significant associations (p < 0.05).

## Data Availability

Unavailable

## Footnotes

1 Republic of Türkiye Ministry of Health, General Directorate of Emergency Health Services (2023). Helicopter ambulance [online]. Website https://acilafet.saglik.gov.tr/TR-78201/helikopter-ambulans.html [accessed 15 October 2025].

2 World Health Organization (2025). WHO guidelines on meningitis diagnosis, treatment and care [online]. Website https://www.who.int/publications/i/item/9789240108042 [accessed 25 March 2026].
